# Supplementary material for: Identification of photoperiod-induced specific miRNAs in the adrenal glands of Sunite sheep (Ovis aries)
Source: Front Vet Sci. 2022 Jul 22;9:888207. doi: 10.3389/fvets.2022.888207 (PMC9354845; doi:10.3389/fvets.2022.888207)
Supplement: Supplementary file 1 [file Data_Sheet_1.ZIP › Supplementary file/miRDeep2 file.zip!/miRDeep2 file/Know_Structure/SP42A1/oar-mir-374a.pdf]

miRBase precursor : oar-mir-374a
Total read count : 18684
oar-miR-374a read count : 15353
remaining reads : 3331

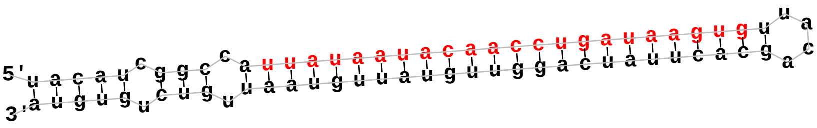

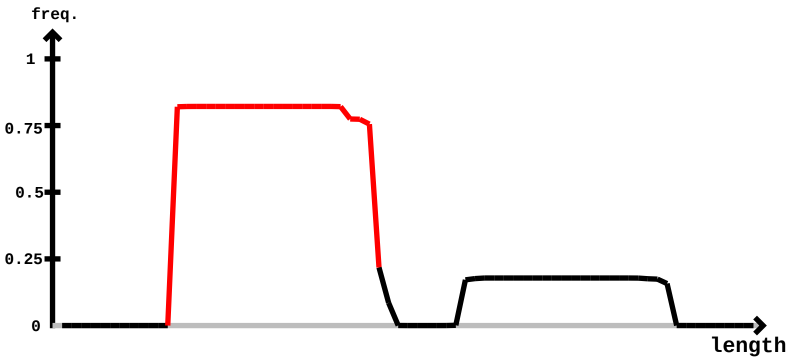


```
                  oar-miR-374a
5'- uacaucggccauuauaauacaaccugauaaguguuacagcacuuaucagguuguauuguaauugucugugua  -3'    exp
    (((((.(((.((((((((((((((((((((((((....)))))))))))))))))))))))).))).)))))   reads      mm         sample
    ...........uuauaauacaacAugau............................................   1          1          seq
    ...........uuauaauacaaccugau............................................   15         0          seq
    ...........uuauaauacaaccugGua...........................................   3          1          seq
    ...........uGauaauacaaccugaua...........................................   1          1          seq
    ...........uuauUauacaaccugaua...........................................   4          1          seq
    ...........uuauaauacaUccugaua...........................................   1          1          seq
    ...........uuauaauacaaccugauU...........................................   20         1          seq
    ...........uuaAaauacaaccugaua...........................................   5          1          seq
    ...........uuauaauacaaccugaua...........................................   816        0          seq
    ...........uCauaauacaaccugaua...........................................   3          1          seq
    ...........Cuauaauacaaccugaua...........................................   3          1          seq
    ...........uuaCaauacaaccugaua...........................................   8          1          seq
    ...........uuauaauacaaccugauaa..........................................   18         0          seq
    ...........uuaCaauacaaccugauaa..........................................   1          1          seq
    ...........uuaCaauacaaccugauaag.........................................   2          1          seq
    ...........uuauaauacaaccugauaag.........................................   337        0          seq
    ...........uGauaauacaaccugauaag.........................................   1          1          seq
    ...........uuauaauacaaccugauaagu........................................   9954       0          seq
    ...........uuaCaauacaaccugauaagu........................................   72         1          seq
    ...........uGauaauacaaccugauaagu........................................   11         1          seq
    ...........uuauaauacaaccugauaagug.......................................   2455       0          seq
    ...........uGauaauacaaccugauaagug.......................................   2          1          seq
    ...........uuaCaauacaaccugauaagug.......................................   15         1          seq
    ...........uuauaauacaaccugauaagugu......................................   1576       0          seq
    ...........uuauaauacaaccugauaaguguu.....................................   4          0          seq
    ............uauaauacaaccugauaagu........................................   7          0          seq
    ............uauaauacaaccugauaagug.......................................   3          0          seq
    ............uauaauacaaccugauaagugu......................................   10         0          seq
    ............uauaauacaaccugauaaguguu.....................................   1          0          seq
    .............auaauacaaccugauaagu........................................   2          0          seq
    .............auaauacaaccugauaagugu......................................   1          0          seq
    ..............uaauacaaccugauaagugu......................................   1          0          seq
    .......................................cacuuaucagguuguauuguaa...........   1          0          seq
    ........................................acuuaucagguuguauugu.............   1          0          seq
    ........................................acuuaucagguuguauuAua............   2          1          seq
```

oar-miR-374a

```
uacaucggccauuauaauacaaccugauaagugguuacagcacuuaucagguuguauuguaauugucugugua
........................................acuuaucagguuguauugua............     2      0      seq
........................................acuuaucagguuguauuguaa...........     1      0      seq
........................................acuuaucagguuguauuguaau..........     6      0      seq
........................................acuuaucagguuguauuguaauu.........     5      0      seq
.........................................cuuaucagguuguauugu.............     4      0      seq
.........................................cuuaucagguuAuauugua............     1      1      seq
.........................................cuuaucagguuguauugua............     41     0      seq
.........................................cuuCucagguuguauugua............     1      1      seq
.........................................cuuaucagguuguauuguaa...........     25     0      seq
.........................................cuuaucagguuAuauuguaau..........     1      1      seq
.........................................cuuaucagguuguauuguaau..........     292    0      seq
.........................................cuuaucagguuguauuAuaau..........     2      1      seq
.........................................cuuaucagguuAuauuguaauu.........     1      1      seq
.........................................cuuaucagguuguauuguaauu.........     2817   0      seq
..........................................uuaucagguuguauuguaau..........     8      0      seq
..........................................uuaucagguuguauuguaauu.........     79     0      seq
...........................................uaucagguuguauuguaauu.........     38     0      seq
...........................................uaucagguuguauuguaauug........     1      0      seq
...........................................uaucagguuguauuguaauugu.......     2      0      seq
```
